# Phospholipid biosynthesis regulation for improving pigment production by *Monascus* in response to ammonium chloride stress

**DOI:** 10.1128/aem.01146-24

**Published:** 2024-09-17

**Authors:** Xiaofei Jiang, Xiya Hong, Zhulin Wang, Jun Liu, Haiyan Zhong, Jiali Ren, Bo Zhou

**Affiliations:** 1 Hunan Key Laboratory of Forestry Edible Sources Safety and Processing, Changsha, China; 2 School of Food Science and Engineering, Central South University of Forestry and Technology, Changsha, China; The Pennsylvania State University, University Park, Pennsylvania, USA

**Keywords:** *Monascus *pigments, membrane lipid homeostasis, ergosterol, CDP-DG pathway, Kennedy pathway

## Abstract

In the actual industrial production process, the efficient biosynthesis and secretion of *Monascus* pigments (MPs) tend to take place under abiotic stresses, which often result in an imbalance of cell homeostasis. The present study aimed to thoroughly describe the changes in lipid profiles in *Monascus purpureus* by absolute quantitative lipidomics and tandem mass tag-based quantitative proteomics. The results showed that ammonium chloride stress (15 g/L) increased MP production while inhibiting ergosterol biosynthesis, leading to an imbalance in membrane lipid homeostasis in *Monascus*. In response to the imbalance of lipid homeostasis, the regulation mechanism of phospholipids in *Monascus* was implemented, including the inhibition of lysophospholipids production, maintenance of the ratio of PC/PE, and improvement of the biosynthesis of phosphatidylglycerol, phosphatidylserine, and cardiolipin with high saturated and long carbon chain fatty acids through the CDP-DG pathway rather than the Kennedy pathway. The inhibition of lysophospholipid biosynthesis was attributed to the upregulated expression of protein and its gene related to lysophospholipase NTE1, while maintenance of the PC/PE ratio was achieved by the upregulated expression of protein and its gene related to CTP: phosphoethanolamine cytidylyltransferase and phosphatidylethanolamine N-methyltransferase in the Kennedy pathway. These findings provide insights into the regulation mechanism of MP biosynthesis from new perspectives.

**IMPORTANCE:**

*Monascus* is important in food microbiology as it produces natural colorants known as *Monascus* pigments (MPs). The industrial production of MPs has been achieved by liquid fermentation, in which the nitrogen source (especially ammonium chloride) is a key nutritional parameter. Previous studies have investigated the regulatory mechanisms of substance and energy metabolism, as well as the cross-protective mechanisms in *Monascus* in response to ammonium chloride stress. Our research in this work demonstrated that ammonium chloride stress also caused an imbalance of membrane lipid homeostasis in *Monascus* due to the inhibition of ergosterol biosynthesis. We found that the regulation mechanism of phospholipids in *Monascus* was implemented, including inhibition of lysophospholipids production, maintenance of the ratio of PC/PE, and improvement of biosynthesis of phosphatidylglycerol, phosphatidylserine, and cardiolipin with high saturated and long carbon chain fatty acids through the CDP-DG pathway. These findings further refine the regulatory mechanisms of MP production and secretion.

## INTRODUCTION

Diverse ingredients (such as enzymes, organic acids, colorants, and polyunsaturated fatty acids) used during food processing are obtained from fungi through industrial fermentation (1). To produce the desired bioproduct as early as possible with the highest possible rate and yield in a consistent manner, in the simplest and cheapest possible way, the substrates used in industrial fermentation vary widely with any materials that can support microbial growth (2). These readily available and inexpensive substrates may be an abiotic stress for microbial cells (3). However, abiotic stress can cause various damages to the microbial cell, the most typical of these is an imbalance in cell membrane homeostasis (4). To ensure cell growth and efficiently produce target metabolites, microorganisms must employ a number of ways to balance cell membrane homeostasis, such as the change in lipid components and their contents in the cell membrane (4, 5).

*Monascus* pigments (MPs), which are produced by various *Monascus* species, have been used as natural microbial-derived food colorant in East Asia, particularly in China and Japan (6). Recently, as MPs, likely *Monascus* yellow pigments (MYPs) and orange pigments (MOPs), have shown good antibacterial and anti-inflammatory activities, more and more attention has been paid to the secondary metabolism and development of *Monascus* spp. (7). However, the production of bioactive secondary metabolites from *Monascus* is closely related not only to the *Monascus* strain but also to unique culture conditions (3, 8, 9). Likely, high concentrations of ammonium chloride could promote and inhibit MPs production for *Monascus anka* mutant MYM (10) and *Monascus purpureus* (CICC 40270) (11), respectively. It is interesting that the abiotic stress caused by ammonium chloride stimulated formation of cross-protection mechanism in *Monascus purpureus* (CICC 40270) (12). As we know, MPs belong to intracellular secondary metabolites, and their biosynthesis and secretion are affected by the cell membrane integrity (including permeability and fluidity) (13), which was mainly determined by the change in lipid profiles and its content in the cell membrane, especially phospholipids (14). More importantly, the biosynthesis of MPs and phospholipids has a common initiator, acetyl-CoA. Although the biochemical basis of phospholipid biosynthesis is relatively well understood, much less is known about how biosynthesis is regulated, particularly how different classes and isoforms are made in order to adapt to high concentrations of ammonium chloride for *Monascus* polyketides. Therefore, elucidating the change in phospholipid profiles and its content in *Monascus* under ammonium salt stress is very important to guide the MPs production.

Hence, according to the changes in the profiles of phospholipids and proteome, the mechanism of phospholipids biosynthesis regulating MPs production in response to high-ammonium chloride stress in *Monascus* has been elucidated using absolute quantitative lipidomics and tandem mass tags (TMT) proteomics approach in combination with bioinformatics analysis in the present work. This work will provide a theoretical basis and practical guidance for the optimized production of MPs with high efficiency and high yield.

## RESULTS

### Effect of ammonium chloride on MPs and citrinin production

**Fig 1:**
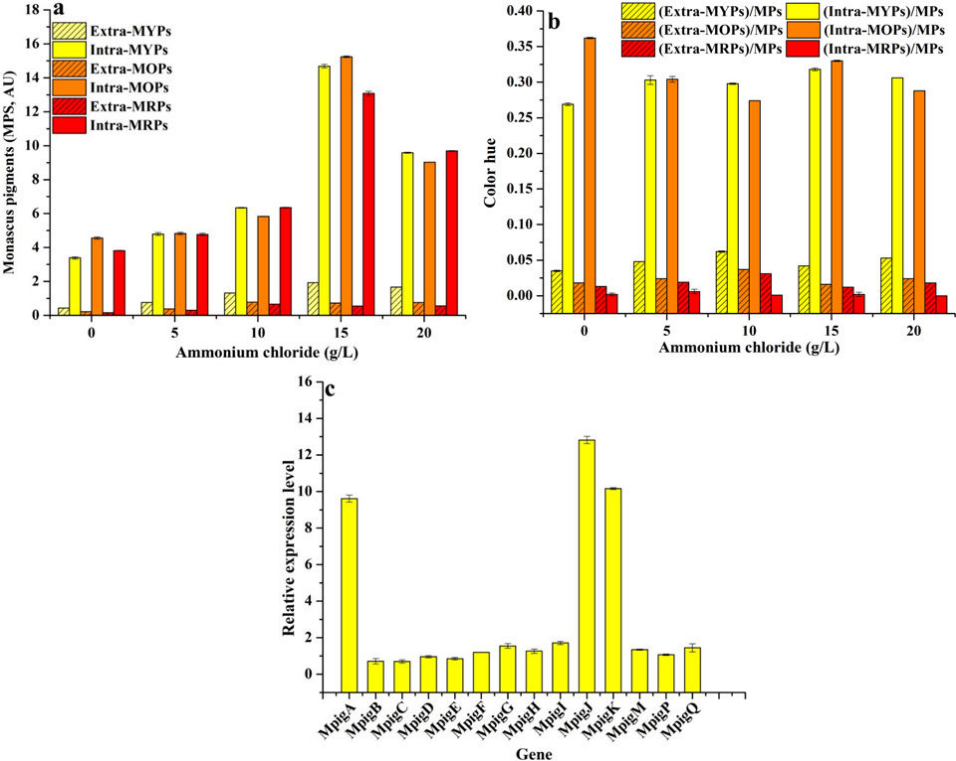
Effect of ammonium chloride on *Monascus* pigments production. (a) MPs production; (b) color hue; (c) expression level of genes related to MPs biosynthesis by qRT-PCR.

The increase in the production of extra/intra-MPs by *Monascus* caused by ammonium chloride (15 g/L) was ascribed to the upregulated expression of genes *MpigA*, *MpigJ,* and *MpigK* (Fig. 1). As shown in Fig. 1a, extra/intra-MYPs, extra/intra-MOPs, and extra/intra-*Monascus* red pigments (MRPs) were 1.93/14.69, 0.73/15.24, and 0.55/13.08 AU/mL under 15 g/L of ammonium chloride, respectively; it was 4.40/4.33 times, 3.31/3.34 times, and 3.43/3.43 times that of ammonium chloride (0 g/L). Extra/intra-MYPs, extra/intra-MOPs, and extra/intra-MRPs accounted for 4.2%/20%, 1.6%/33%, and 1.2%/0.28% of total MPs, which was 1.20/1.18 times, 0.90/0.91 times, and 0.93/0.93 times than that of ammonium chloride (0 g/L) (Fig. 1b). These results indicated that 15 g/L of ammonium chloride promoted the production of extra/intra-MPs. The expression of genes *MpigA*, *MpigJ,* and *MpigK* was upregulated by 9.61, 12.82, and 10.16 times in *Monascus* in response to 15 g/L of ammonium chloride (Fig. 1c), respectively. These results further indicated the promotion of MP production may be attributed to the upregulated expression of *MpigA*, *MpigJ,* and *MpigK*.

It is interesting that citrinin was not detected regardless of the concentration of ammonium chloride added in this work.

### Effect of ammonium chloride on cell membrane homeostasis in *Monascus*

**Fig 2:**
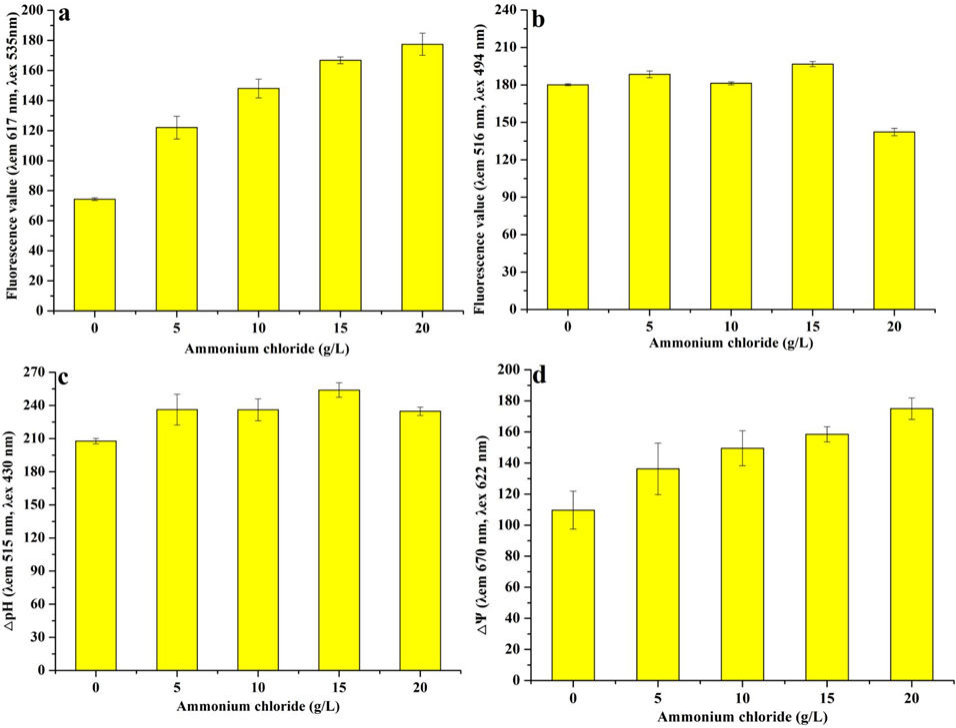
Effect of ammonium chloride on cell membrane homeostasis of *Monascus*. (a) Membrane integrity; (b) Ca^2+^ concentration; (c) pH gradient (△pH); (d) transmembrane potential (△Ψ).

**Fig 3:**
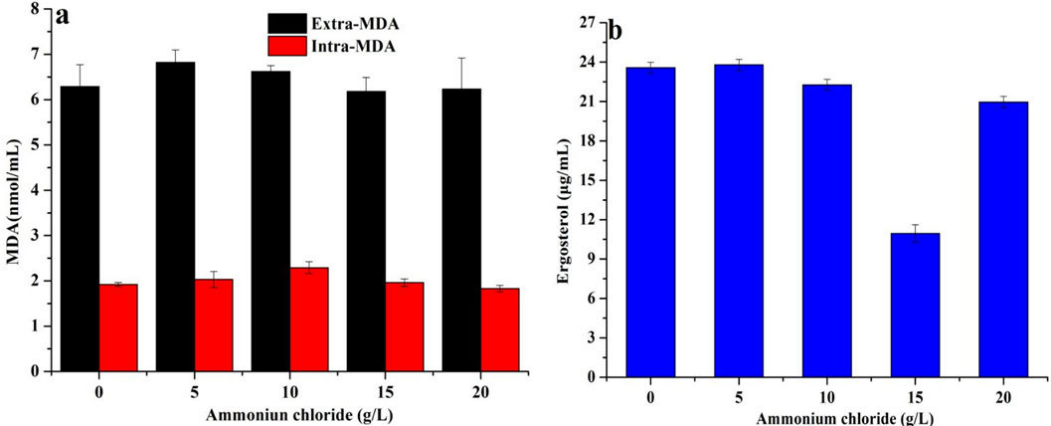
Effect of ammonium chloride on the content of MDA (**a**) and ergosterol (**b**) in *Monascus.*

The imbalance of homeostasis in *Monascus* caused by ammonium chloride (15 g/L) was due to the decrease in ergosterol content (Fig. 2 and 3). The fluorescence values of membrane integrity (Fig. 2a), Ca^2+^ concentration (Fig. 2b), pH gradient (Fig. 2c) and transmembrane potential (Fig. 2d) in *Monascus,* which was cultured with 15 g/L of ammonium chloride, were 2.24 times, 1.09 times, 1.22 times, and 1.45 times than that of 0 g/L of ammonium chloride, respectively. These results showed that 15 g/L of ammonium chloride caused a decrease in the fluidity and permeability of *Monascus* cell membranes. Meanwhile, the contents of extracellular malonaldehyde (MDA) (6.18 nmol/mL) (Fig. 3a), total MDA (8.14 nmol/mL) (Fig. 3a), and ergosterol (10.95 µg/mL) (Fig. 3b) were 0.98 times, 0.99 times, and 0.46 times than that of ammonium chloride (0 g/L), respectively. These results further indicate that it is not oxidative damage but the inhibition of ergosterol biosynthesis caused an imbalance of homeostasis*,* which ultimately led to the imbalance of intra/extra pH gradient and transmembrane potential and an increase in Ca^2+^ concentration in *Monascus*.

### Effect of ammonium chloride on lipid profiles in *Monascus*

**Fig 4:**
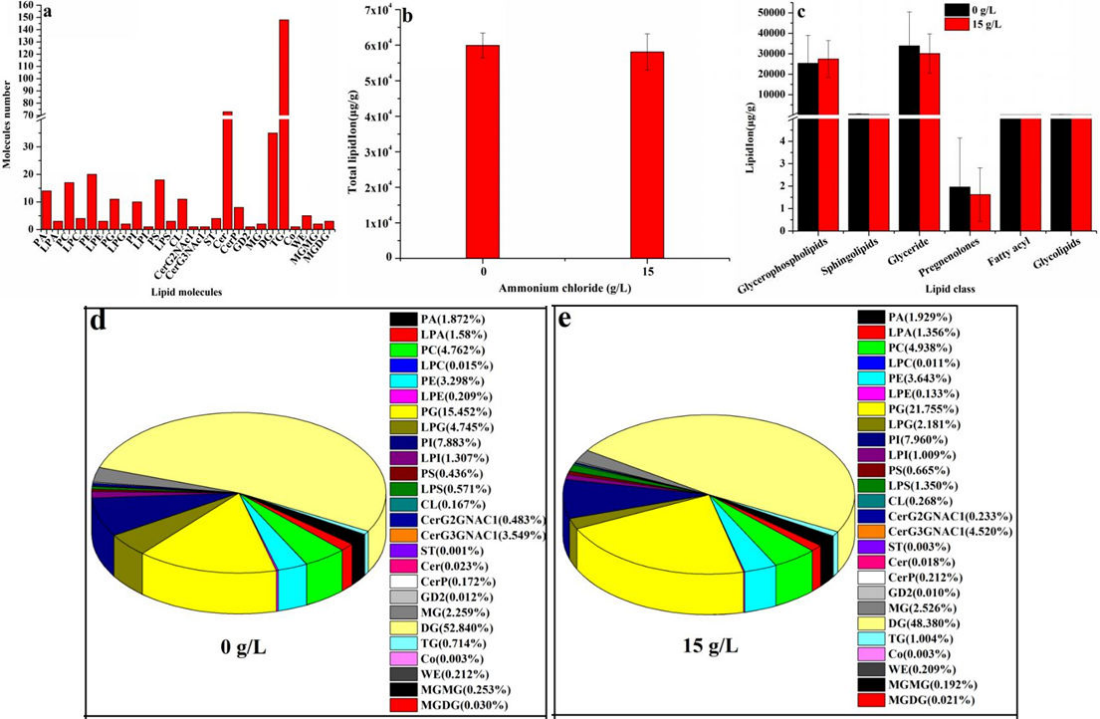
Effect of ammonium chloride on lipid composition and content in *Monascus*. (a) Number of lipid molecules; (b) total lipidIon; (c) lipid class; (d) lipid composition in *Monascus* cultured with ammonium chloride 0 g/L; and (e) lipid composition in *Monascus* cultured with ammonium chloride 15 g/L.

Ammonium chloride (15 g/L) promoted the biosynthesis of glycerophospholipids and significantly inhibited the biosynthesis of sphingolipids, glyceride, pregnenolones, and glycolipids (Fig. 4). According to the LIPID MAPS system nomenclature (15), there are 401 kinds of lipid molecules in *Monascus purpureus* (CICC 5013), which belong to 26 subspecies, including 13 kinds of glycerophospholipids, 6 kinds of sphingolipids, 3 kinds of glycerides, 2 kinds of glycolipids, 1 kind of fatty acyl, and 1 kind of progesterone (Fig. 4a). The total lipid content (58,086.37 µg/g) under 15 g/L of ammonium chloride was 0.97 times than that of ammonium chloride (0 g/L) (Fig. 4b), and the contents of glycerophospholipids, sphingolipids, glycerides, pregnenolones, fatty acyl, and glycolipids were 1.08 times, 0.67 times, 0.89 times, 0.83 times, 0.95 times, and 0.73 times than that of ammonium chloride (0 g/L) (Fig. 4c), respectively. Under 0/15 g/L of ammonium chloride, the top five percentage of lipids in *Monascus* was all diacylglycerol (DG), phosphatidylglycerol (PG), phosphatidylinositol (PI), and phosphatidylcholine (PC) (Fig. 4e and d), which accounted for 52.84%/48.38%, 15.45%/21.76%, 7.88%/7.96%, 4.76%/4.94%, and 3.30%/3.64%, respectively. The above results showed that ammonium chloride (15 g/L) does not have a significant influence on the types of total lipids and their contents, in addition to phospholipids; however, it significantly reduced and increased the ratio of DG and PG in *Monascus*, respectively.

### Effect of ammonium chloride on species, saturation, and chain length of glycerides

**Fig 5:**
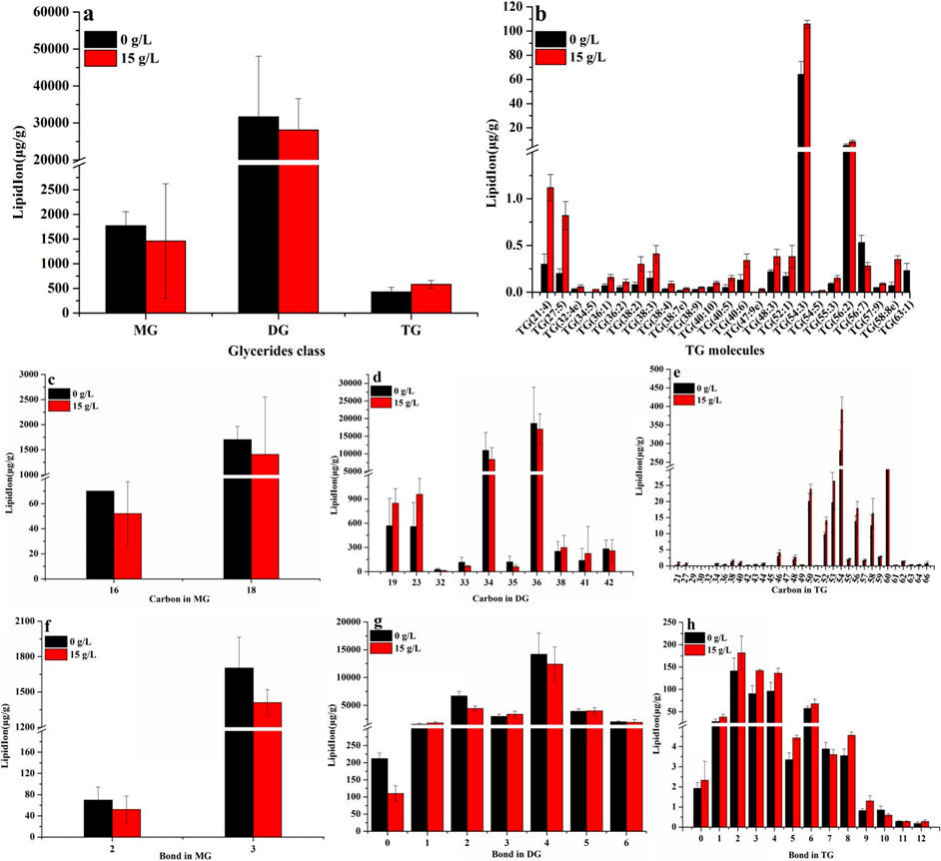
Effect of ammonium chloride on species, saturation, and chain length of glycerides. (a) Glyceride class; (b) TG species; (c) chain length of MG; (d) chain length of DG; (e) chain length of TG; (f) saturation of MG; (g) saturation of DG; (h) saturation of TG.

Ammonium chloride (15 g/L) inhibited the biosynthesis of DG and monoglyceride (MG) and promoted the biosynthesis of triglyceride (TG) with high saturation and long chain (Fig. 5). The contents of MG, DG, and TG in *Monascus* under ammonium chloride (15 g/L) were 0.82 times, 0.89 times, and 1.36 times than those of ammonium chloride (0 g/L ) (Fig. 5a), respectively. The contents of TG with long carbon chain have been significantly increased in *Monascu*s in response to 15 g/L of ammonium chloride (Fig. 5b and e). Likely, the content of TG(38:2), TG(27:5), TG(58:8e), TG(34:5), and TG(47:9e) was 3.92 times, 4.08 times, 4.81 times, 5.88 times, and 7.06 times than that of ammonium chloride (0 g/L) (Fig. 5b), respectively. Meantime, the content of TG with a saturation of 3, 4, 5, 8, and 9 has been significantly increased under 15 g/L of ammonium chloride, which was 1.57 times, 1.42 times, 1.32 times, 1.28 times, and 1.58 times than that of ammonium chloride (0 g/L) (Fig. 5h), respectively. These results show that the biosynthesis of TG with high saturation and long chain has been increased in the present work.

### Effect of ammonium chloride on species, saturation, and chain length of glycerophospholipids

**Fig 6:**
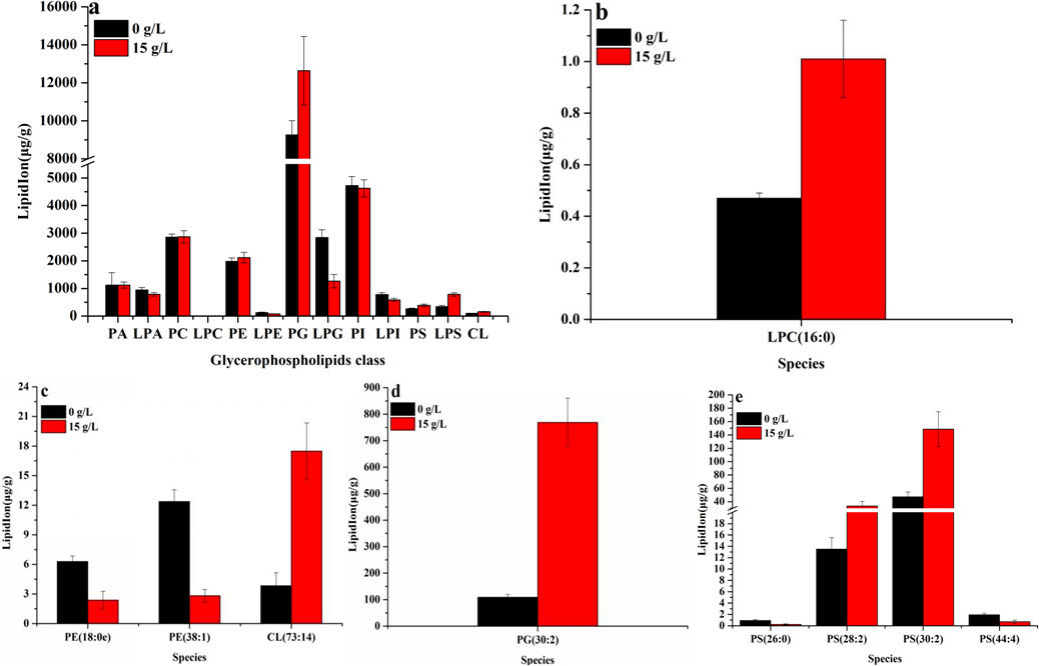
Effect of ammonium chloride on the content of glycerophospholipid. (a) Glycerophospholipid class; (b) LPC; (c) PE and CL; (d) PG; (e) PS.

Ammonium chloride (15 g/L) increased the contents of PG, phosphatidylserine (PS), phosphatidylethanol (PE), and cardiolipin (CL), while decreasing the contents of lysophospholipids (except for LPS) and had no significant influence on the contents of phosphatidic acid (PA) and PC (Fig. 6). One kind of CL and six kinds of phospholipids and their lysophospholipids were found, and 15 g/L of ammonium chloride significantly promoted the biosynthesis of PG, PS, LPS, and CL (Fig. 6a); likely, the content of PG under 15 g/L ammonium chloride (12,636.66 µg/g) was 1.36 times than that of 0 g/L of ammonium chloride (9,262.04 µg/g) (Fig. 6a). The content of PG (30:2), PS(28:2), PS(30:2), and CL(73:14) was 7.04 times (Fig. 6d), 2.48 times (Fig. 6e), 3.15 times (Fig. 6e), and 4.56 times (Fig. 6c) than that of ammonium chloride (0 g/L), respectively. The above results showed that ammonium chloride mainly promoted the biosynthesis of PG, PE, PS, LPS, and CL.

**Fig 7:**
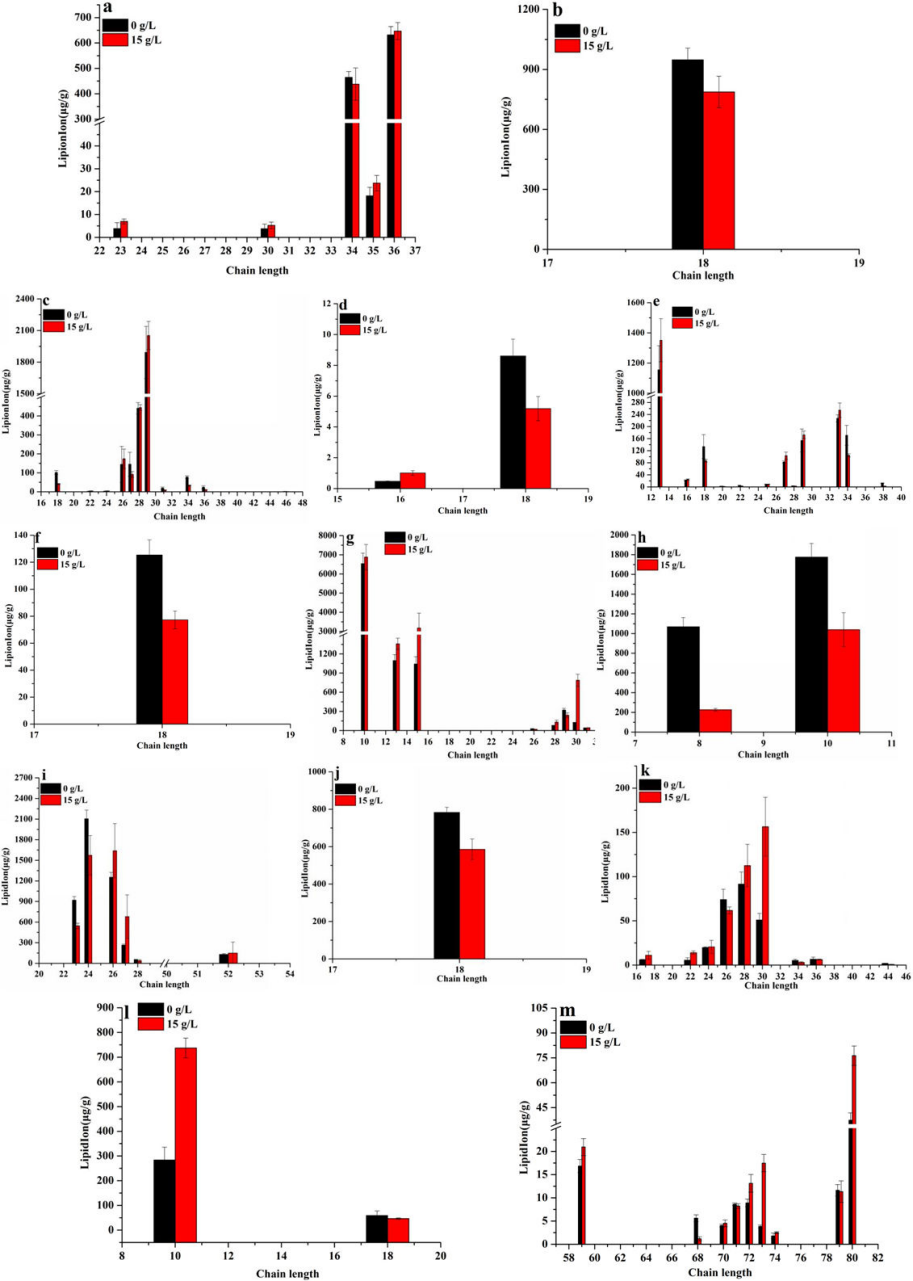
Effect of ammonium chloride on the content of glycerophospholipids with different chain lengths. (a) PA; (b) LPA; (c) PC; (d) LPC; (e) PE; (f) LPE; (g) PG; (h) LPG; (i) PI; (j) LPI; (k) PS; (l) LPS; (m) CL.

**Fig 8:**
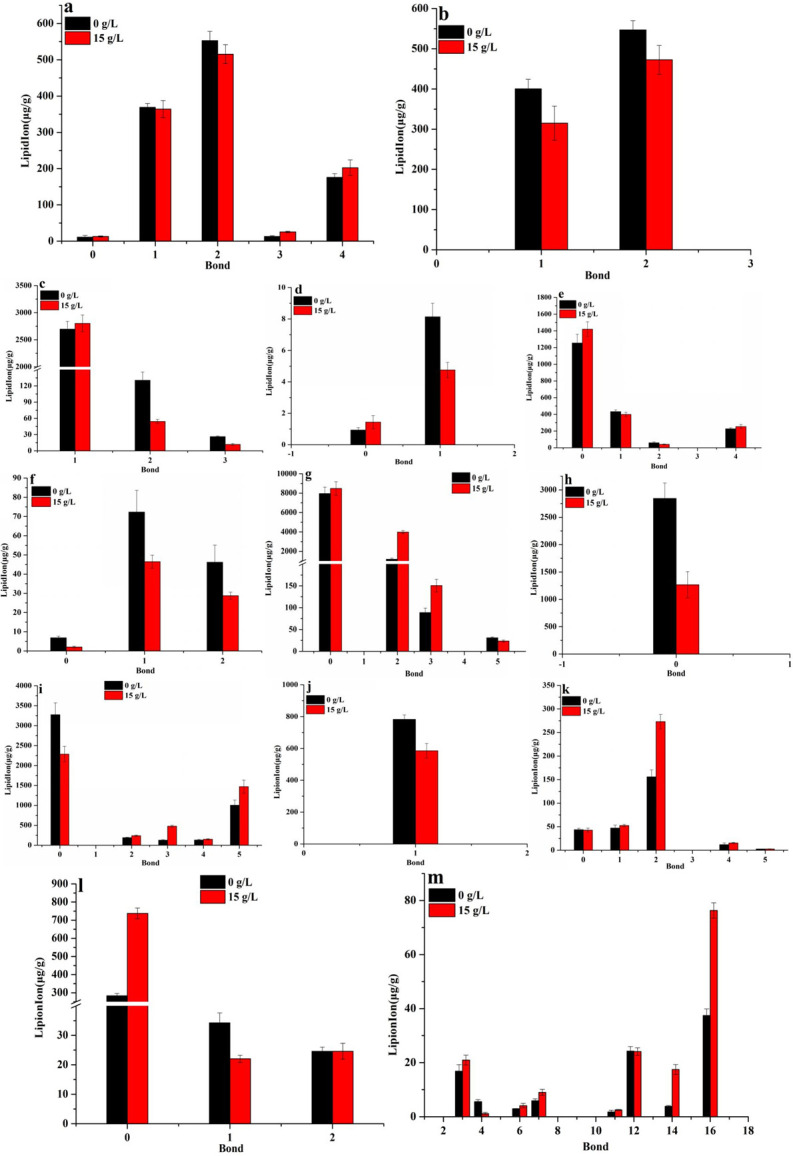
Effect of ammonium chloride on the content of glycerophospholipids with different saturation. (a) PA; (b) LPA; (c) PC; (d) LPC; (e) PE; (f) LPE; (g) PG; (h) LPG; (i) PI; (j) LPI; (k) PS; (l) LPS; (m) CL.

As far as chain length was concerned, ammonium chloride (15 g/L) benefits the biosynthesis of PG, PS, PE, and CL with long chain fatty acids in *Monascus* (Fig. 7) . In this work, the content in PA with chain length of 34 has been decreased by 0.94 times; however, the PA content with others chain length was increased by 1.02–1.83-fold (Fig. 7a). The contents in PC with different chain lengths have been decreased by 0.4–0.6-fold, while others were not obvious (Fig. 8c). The contents in PE with different chain lengths have been decreased by 0.23–0.65-fold, while others were not obvious (Fig. 8e). The contents in PG with chain lengths of 15, 28, and 30 have been increased by 3.05-fold, 1.68-fold, and 6.51-fold, respectively, while those of 26 and 29 have been decreased by 0.71- and 0.75-fold; others were not obvious (Fig. 8g). The contents in PI with chain lengths of 26, 27, and 52 has been increased by 1.31-, 2.58-, and 1.14-fold, respectively, while those of 23, 24, and 28 have been decreased by 0.59-, 0.74-, and 0.71-fold (Fig. 8i), respectively. The contents in PS with chain lengths of 17, 22, 24, 28, and 30 have been increased by 1.84-, 2.55-, 1.04-, 1.23-, and 3.07-fold, respectively, while those of 26, 34, 36, and 44 have been decreased by 0.83-, 0.57-, 0.95-, and 0.37-fold (Fig. 8k), respectively. The contents in CL with chain lengths of 68, 71, and 79 have been decreased by 0.21-, 0.96-, and 0.98-fold, respectively, while those of other chain length have been increased by 1.12–4.55-fold (Fig. 8m). The content in LPC with a chain length of 16 and LPS with a chain length of 10 has been increased by 2.15-fold (Fig. 7c) and 2.6-fold (Fig. 7i), respectively; the contents of other lysophospholipids with different chain lengths were decreased by 0.21–0.75-fold. These results showed that ammonium chloride (15 g/L) promoted the biosynthesis of phospholipids with long-chain fatty acids.

In terms of saturation, ammonium chloride mainly promoted the biosynthesis of PG, PS, and CL with high saturation (Fig. 8). In this work, the contents in PA with bonds of 1 and 2 have decreased by 0.99- and 0.93-fold, respectively, and others have increased by 1.17–1.94-fold (Fig. 8a). The contents in PC with a bond of 1 have increased by 1.04-fold, and the others have decreased by 0.42–0.45-fold (Fig. 8c). The contents in PE with bonds of 0 and 4 have increased by 1.13- and 1.12-fold, respectively, while those with bonds of 1 and 2 were decreased by 0.92- and 0.69-fold (Fig. 8e), respectively. The content in PG with a bond of 5 has decreased by 0.79-fold, while others have increased by 1.07–3.35-fold (Fig. 8g). The content in PI with a bond of 0 has decreased by 0.70-fold, while others have increased by 1.14–3.79-fold (Fig. 8i). The content in PS with a bond of 0 has decreased by 0.98-fold, while others have all increased by 1.00–1.75-fold (Fig. 8k). The content in CL with bonds of 3, 6, 7, 14, and 16 has increased by 1.24-, 1.39-, 1.52-, 1.4-, 4.55-, and 2.04-fold, respectively, while those with bonds of 4 and 12 have decreased by 0.21- and 0.99-fold (Fig. 8m), respectively. The contents in LPC with a bond of 0 and LPS with a bond of 0 have increased by 1.54-fold (Fig. 8c) and 2.6-fold (Fig. 8i), respectively; the contents of other lysophospholipids with different saturation were decreased by 0.3–0.75-fold. These results showed that ammonium chloride mainly promoted the biosynthesis of PG, PS, and CL with high saturation, while the opposite was true for PE.

### Effect of ammonium chloride on the expression of proteins and genes related to lipid metabolism

**Fig 9:**
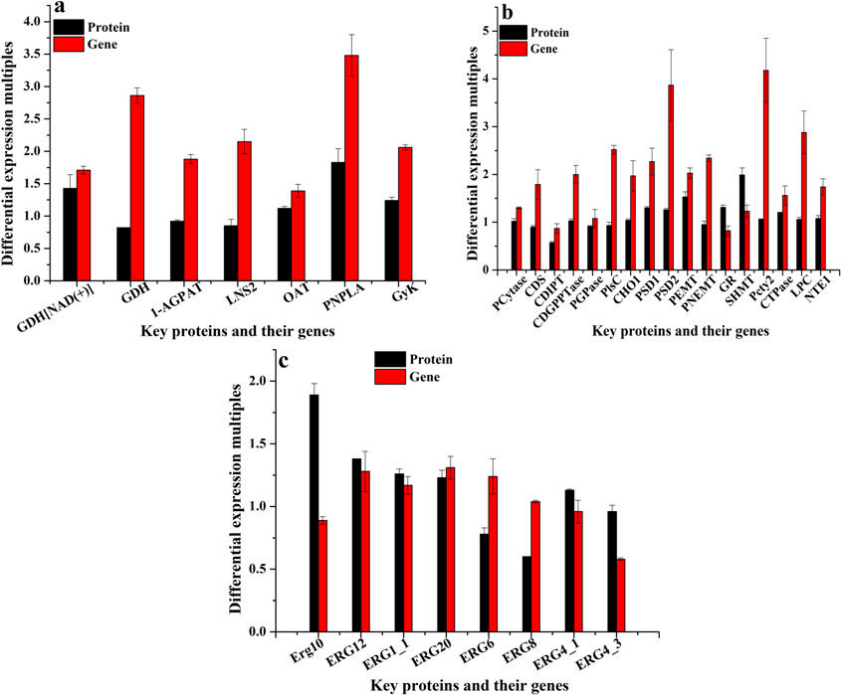
Effect of ammonium chloride on the expression levels of key proteins and their genes related to lipid metabolism. (a) Glyceride; (b) glycerophospholipid; (c) ergosterol. GDH [NAD(+)], glycerol-3-phosphate dehydrogenase; GDH, glycerol-3-phosphate dehydrogenase; 1-AGPAT, 1-acyl-sn-glycerol-3-phosphate acyltransferase; LNS2, LNS2 domain-containing protein; OAT, O-acyltransferase; PNPLA, patatin-like phospholipase domain-containing protein; GyK, glycerol kinase; PCytase, phosphatidate cytidylyltransferase; CDS, CDP-diacylglycerol synthase; CDIPT, CDP-diacylglycerol-inositol 3-phosphatidyltransferase; CDGPPTase, CDP-diacylglycerol-glycerol-3-phosphate 3-phosphatidyltransferase; PGPase, phosphatidylglycerophosphatase; PlsC, PlsC domain-containing protein; CHO1, CDP-diacylglycerol-serine O-phosphatidyltransferase; PSD1, phosphatidylserine decarboxylase proenzyme 1; PSD2, phosphatidylserine decarboxylase proenzyme 2; PEMT, phosphatidylethanolamine N-methyltransferase; PNEMT, phosphatidyl-N-methylethanolamine N-methyltransferase; GR, glyoxylate reductase; SHMT, serine hydroxymethyltransferase; Pcty2, CTP:phosphoethanolamine cytidylyltransferase; CTPase, CTP_transf_like domain-containing protein; LPC, lysophospholipase; NTE1, lysophospholipase NTE1; Erg10, Acetyl-CoA C-acetyltransferase; ERG12, mevalonate kinase; ERG1_1, squalene epoxidase; ERG20, farnesyl pyrophosphate synthetase; ERG6, sterol 24-C-methyltransferase; ERG8, phosphomevalonate kinase; and ERG4_1 and ERG4_3, C-24(28)) sterol reductase.

Ammonium chloride (15 g/L) could increase the expression of proteins and genes related to the biosynthesis of TG and glycerol phospholipids in *Monascus* (Fig. 9). Ammonium chloride promoted the expression of proteins/genes of triglyceride lipase and glycerol kinase in the triglyceride biosynthesis pathway, which has been upregulated by 1.83/1.24 times and 3.48/2.06 times (Fig. 9a), respectively. In terms of glycerophospholipid biosynthesis, the expression of protein/gene of phosphatidylserine decarboxylase, phosphatidylethanolamine *N*-methyltransferase, glyoxylate reductase, serine hydroxymethyltransferase, and choline cytidyl phosphate transferase has increased under 15 g/L of ammonium chloride, and the protein/gene expression of these key enzymes has been upregulated by 1.30/2.27 times, 1.26/3.87 times, 1.53/2.03 times, 1.31/0.82 times, 1.99/1.23 times, and 1.20/1.56 times (Fig. 9b), respectively. In addition, ammonium chloride could also increase the expression of genes related to lysophospholipase and lysophospholipase NTE1, which were upregulated by 2.88 times and 1.74 times (Fig. 9b), respectively.

**Fig 10:**
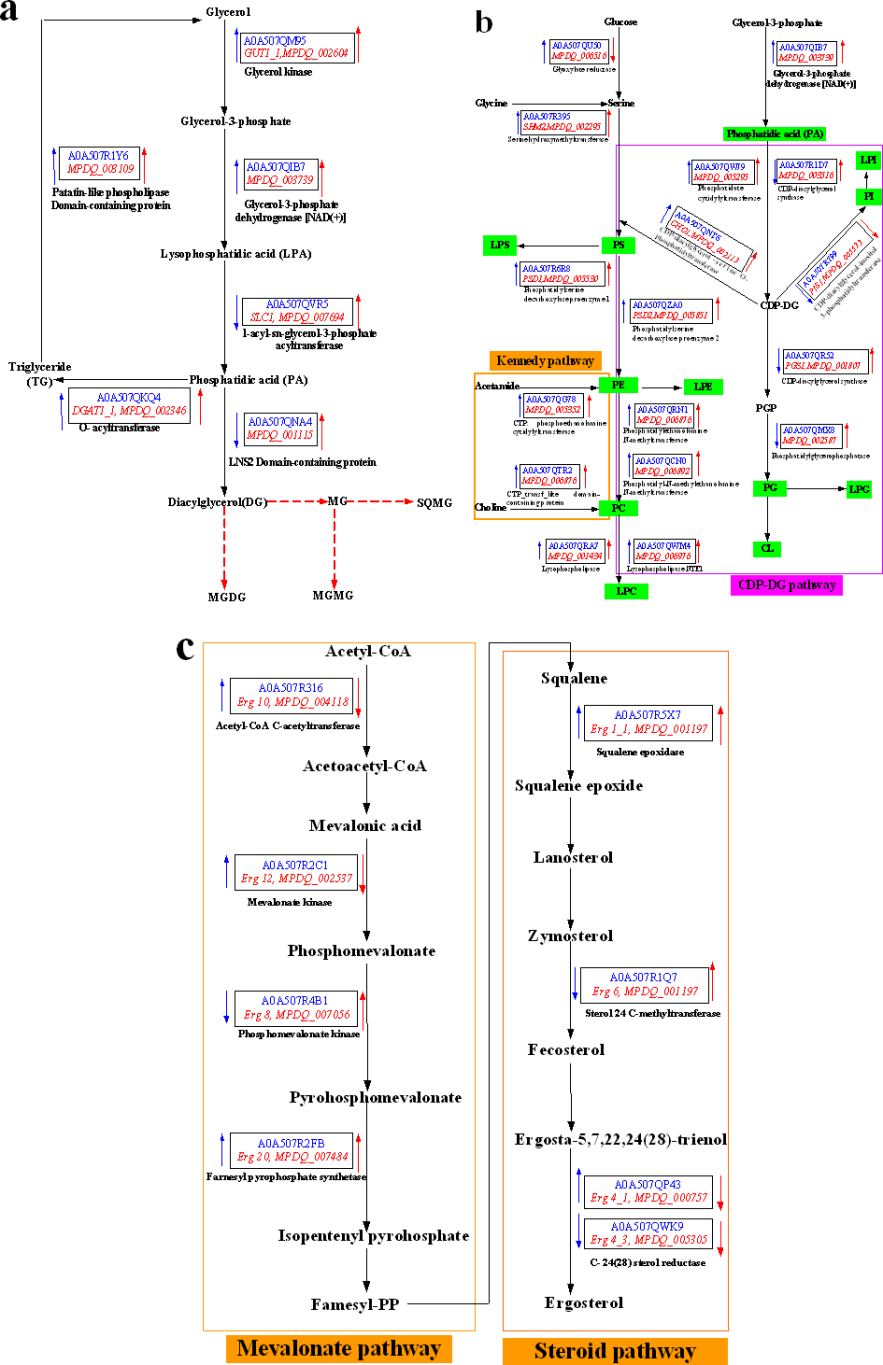
Mechanistic analysis of ammonium salts on biosynthesis of TG (**a**), glycerophospholipid (**b**), and ergosterol (**c**). Blue font indicates relevant differential proteins in proteomics, red italics indicate genes encoding these proteins, and arrows indicate up- or downregulation of expression or transcription.

## DISCUSSION

The main secondary metabolites of *Monascus* are polyketones (including pigments and citrinin), and their biosynthesis is influenced by not only the strain itself but also the culture conditions (8). In the present work, 15 g/L of ammonium chloride promoted the production of extra/intra-MPs (Fig. 1a), which was not consistent with *Monascus purpureus* (CICC 40270) (11) but with *Monascus anka* mutant MYM (10), and increased gene expression of *MpigA* (coding polyketide synthase), *MpigJ* (coding α subunit of fatty acid synthase), and *MpigK* (coding β subunit of fatty acid synthase) (Fig. 1c). As the three most important proteases that regulate the MPs production, which are all closely related to polymeric acetyl-CoA and malonyl-CoA into the first intermediate, tetraketone, of MPs (16), the deletion of these genes could lead to complete inhibition of MP production (17, 18). Therefore, the increase in MP contents may be attributed to the upregulated expression of *MpigA*, *MpigJ,* and *MpigK,* which were all closely related to the promotion initiation of MP production.

*Monascus* polyketones belong to intracellular secondary metabolites, and their biosynthesis and secretion are affected by the cell membrane integrity (including permeability and fluidity) (13). In this work, the increase in intracellular Ca^2+^ content (Fig. 2b), pH gradient (Fig. 2c), and transmembrane potential (Fig. 2d) indicated that the membrane integrity and permeability of *Monascus* were decreased and increased, respectively, leading to an imbalance in *Monascus* membrane homeostasis (Fig. 2 and 3). Theoretically, in response to abiotic stress, cells can produce a large number of superoxide free radicals, which causes membrane lipids to undergo peroxidation and produce MDA, resulting in changes to the structure and function of the cell membrane (19). The unobvious difference in MDA contents (Fig. 3a) and lipidomics data (Fig. 4) in this work indicates that the imbalance of cell membrane homeostasis in *Monascus* was not ascribed to lipid oxidation and composition changes in lipid subclasses but content changes in lipid components, which is consistent with previous reports on *Monascus purpureus* (CICC 40270) (12).

The lipids of the cell membrane are mainly composed of glycerol phospholipids, sphingolipids, and ergosterol in fungi (20). The concentration of ergosterol, a major component of fungal cell membranes, is positively correlated with membrane permeability, and changes in cell membrane permeability are positively correlated with cellular homeostasis (20). Ammonium chloride (15 g/L) caused a decrease in ergosterol content in *Monascus* (Fig. 3b) in this work. This result shows that one of the reasons for the imbalance of *Monascus* membrane homeostasis may be attributed to the suppression of ergosterol biosynthesis in *Monascus*. TMT proteomics data further showed that the expression of proteins, including sterol C-24 methyltransferase (which catalyzes the synthesis of lanosterol into ergosterol) (21) and phosphomevalonate kinase (which catalyzes the synthesis of mevalonate phosphate from mevalonate pyrophosphate) (22), has been decreased in *Monascus* in response to 15 g/L of ammonium chloride (Fig. 9e and 10c). Meantime, the expression of most genes related to ergosterol biosynthesis was downregulated (Fig. 9e), especially for *ERG4* [coding C-24 (28) sterol reductase, catalyzing the conversion of ergosta-5,7,22,24 (28)-tetraenol to ergosterol in the last step of the ergosterol biosynthetic pathway) (23). In the meantime, the biosynthesis promotion for TG (Fig. 5c) and phospholipids (PG, PS, and CL) (Fig. 6c through e) with long carbon chain was also one reason for the fluidity decrease in the *Monascus* cellular membrane. These results indicated that ammonium chloride (15 g/L) inhibited ergosterol production in *Monascus* by downregulating the expression of proteins and genes related to ergosterol biosynthesis, leading to a decrease in cell membrane integrity, which was consistent with previous reports (24).

In response to the environmental stresses, organisms will also constantly seek new metabolic pathways to improve their tolerance by maintaining the integrity of cytoplasmic membrane and then ensure the normal and orderly progress of intracellular biochemical reactions, exchange of information, matter, and energy with the surrounding environment (25). An important factor affecting the integrity of the cell membrane is the change in compositions and contents of lipids in the membrane (26), including the distribution of phospholipid head groups (27), the ratio of saturated and unsaturated lipid acyl chains (28), and the content of ergosterol and sphingolipids (29). In the present work, ammonium chloride (15 g/L) caused an unobvious decrease in total contents of lipids (Fig. 4b), while promoting the biosynthesis of glycerophospholipids (Fig. 4c) [especially for PG, PE, PS, and CL (Fig. 6a)] and inhibiting the biosynthesis of lysophospholipids (except for LPS) (Fig. 6a), sphingolipids, glycerides, pregnenolones [especially for ergosterol (Fig. 3b)], and glycolipids (Fig. 4c). In this work, these results indicate that *Monascus purpureus* CICC 5013 mainly regulated the contents of glycerophospholipid to cope with the changes in membrane homeostasis caused by ammonium chloride stress.

Glycerophospholipids include phospholipids and lysophospholipids. Phospholipid (mainly including PC, PE, PS, PI, PG, and CL) as the basic component of membrane lipids (accounting for more than 50%) is one of the key factors determining membrane fluidity (30), especially for the length and saturation of fatty acid in phospholipid molecules. As described in the above results, ammonium chloride stress inhibited the biosynthesis of DG and MG and promoted the biosynthesis of TG with high saturation and long chain (Fig. 5). The decrease in DG contents, a common precursor for the biosynthesis of PC, PE, and PS (Fig. 10b) (30), in *Monascus* in response to ammonium chloride stress was lower (Fig. 5a). Theoretically, a decrease in DG contents would result in a decrease in the biosynthesis of PC, PE, and PS, but the opposite was true: the biosynthesis of PG, PE, PS, and CL (Fig. 6a) has been improved, and there has been no significant influence on the biosynthesis of PC and PA (Fig. 6a). These results indicated that phospholipid biosynthesis in *Monascus* in response to ammonium chloride stress was mainly via the CDP-DG pathway (from PA to CDP-DG) (14) rather than the Kennedy pathway (from PA to DG) (30). Even though the biosynthesis promotion of fatty acid with long carbon chain in PG (Fig. 7g), PS (Fig. 7k), and CL (Fig. 7m) decreased the membrane fluidity (Fig. 2a), it improved the fluidity by increasing the saturation of fatty acid in PG (Fig. 8g), PS (Fig. 8k), and CL(Fig. 8m).

Interestingly, even though the proteomics data were significantly different, transcriptional expression of genes involved in the TG biosynthesis pathway was upregulated (Fig. 9a and 10b). This may account for the greater conversion of DG and MG to TG with high saturation (Fig. 5) and also indicates that DG could enhance the fluidity of phospholipid to relieve the imbalance of *Monascus* cell membrane homeostasis. In the meantime, the decreased content of DG and MG (Fig. 5a) also indicated that the biosynthesis of sphingolipids and glycolipids in cytomembrane has been inhibited (Fig. 4b, 9, and 10b ) (26).

The phospholipids of the plasma membrane in *Monascus* are mainly PC and PE. The contents and species change of which are mainly related to the cytomembrane fluidity of cell response to environmental stresses (31). The distribution of PC and PE on the membrane is an important regulator for plasma membrane integrity and transmembrane transport of substances, and the decrease in the ratio of PC/PE will reduce the integrity of the plasma membrane and then enhance the permeability of the cell membrane (32). In this work, the contents of PC (Fig. 6a) and PE (Fig. 6c) have decreased, with a PC/PE ratio of 1.44 (0 g/L) and 1.36 (15 g/L). This indicates that ammonium chloride stress has disrupted the integrity of *Monascus* cytomembrane. Even though ammonium chloride (15 g/L) increased the expression of protein and its gene related to phosphatidylethanolamine N-methyltransferase (PEMT, catalyzed PE to generate PC) (Fig. 9b and 10b), the decrease in the ratio of PC/PE may be attributed to the expression of gene related to lysophospholipase (hydrolyze PC into LPC) (Fig. 6b and 10b). Therefore, the upregulated expression of PEMT played a role in maintaining the balance between PC and PE (33) and maybe a way for *Monascus* to respond to the decrease in cytomembrane integrity caused by 15 g/L of ammonium chloride. These results indicate that the balance of *Monascus* cell membrane homeostasis is mainly regulated by promoting the biosynthesis of phospholipids (including PG, PE, PS, and CL) via the CDP-DG pathway and the Kennedy pathway.

Lysophospholipids are mainly produced by the degradation of phospholipids, which can cause membrane rupture and thus lead to cell hemolysis or necrosis, and lysophospholipids can also be converted into phospholipids by lysophospholipid acyltransferase to avoid damage to cell membranes (34). In this work, the decrease in lysophospholipid content (except for LPS) (Fig. 6a) may be attributed to the upregulated expression of gene related to lysophospholipase NTE1 [complete degradation of lysophospholipids to fatty acids, glycerides, and phospholipid acyl groups (14)] (Fig. 9b and 10b). These results indicate that the upregulated expression of proteins and genes related to lysophospholipase NTE1 may reduce the level of lysophospholipids, which in turn maintain the cell membrane to avoid disruption caused by ammonium chloride (15 g/L).

The mitochondrial membrane has a high content of non-bilayer-forming phospholipids, such as PE and CL, and the loss of these non-bilayer-forming phospholipids will cause damage to mitochondrial function (35, 36). Mitochondria are capable of PE and CL biosynthesis, while the biosynthesis of other phospholipids occurs primarily in the endoplasmic reticulum. In this work, ammonium chloride (15 g/L) could increase the content of PE, PG [especially for PG(30:2) (Fig. 6d)], and CL (Fig. 6a) [especially for CL(73:14) (Fig. 6c)]. As we know, the main source of PE in cells is attributed to the decarboxylation of PS by phosphatidylserine decarboxylase (PSD) located in the inner mitochondrial membrane (37). In this work, the increase in PE contents may be attributed to the upregulated expression of proteins and their genes related to the proenzyme of PSD (Fig. 9b and 10b), which may be a measure to alleviate mitochondrial, morphological, and functional abnormalities caused by ammonium chloride stress (38). It is very interesting to note that ammonium chloride stress significantly inhibited the biosynthesis of PE (18:8e) and PE (38:1) (Fig. 6c). These results indicate that the imbalance in mitochondrial membrane lipid homeostasis may be attributed to the biosynthesis promotion for PE with short chain and low saturation. Meanwhile, the upregulated expression of protein and its gene related to phosphatidylethanolamine N-methyltransferase (catalyzing PE to generate PC) (Fig. 9b and 10b) indicated that PE was more easily converted into PC.

CL, a specific lipid component of mitochondria, is mainly distributed in the inner membrane of mitochondria and can promote the formation of non-bilayer structures to maintain mitochondrial integrity under a low pH environment (39). Meanwhile, CL is necessary to maintain many mitochondrial functions, likely regulating ATP production in the mitochondrial respiratory chain (40). More research has shown that CL could increase ATP levels in the mitochondria by promoting the efficient transfer of electrons from complex III to complex IV (36), improving the efficiency of oxidative phosphorylation in cells and their resistance to adverse conditions, and resisting the toxicity of its own surface-active antibacterial agents (41). In our previous report (12), high concentration ammonium chloride improved the activity and expression of complex I and inhibited the activity of complex III and IV. So, the increase in the contents of CL (Fig. 6a, 7m, and 8m) with long chain and high saturation, especially for CL(73:14) (Fig. 6c), in *Monascus* under ammonium chloride stress may be a measure to stabilize mitochondrial membrane structure, maintain mitochondrial energy conversion, and other functions.

In brief, first, ammonium chloride stress increased the expression of proteins and their genes related to the TG biosynthesis pathway (Fig. 10a), promoted the biosynthesis of glycerol-3-phosphate, and then the contents of TG increased (Fig. 5a) due to the upregulated expression of protein related to glycerol kinase (which catalyzes glycerol to produce glycerol-3-phosphate) (Fig. 9a and 10a). The increase in TG contents is the imbalance of energy homeostasis due to the inhibition of ATP production in *Monascus* in response to ammonium chloride stress (42). Second, ammonium chloride stress promoted the biosynthesis of glycerophospholipids (Fig. 4c) [especially for PG, PE, PS, and CL (Fig. 6a)] and inhibited the biosynthesis of lysophospholipids (except for LPS) (Fig. 6a) via the CDP-DG pathway because of upregulated expression of genes coding cytidyl phosphate transferase, CDP-diacylglycerol synthase, and CDP-diacylglycerol-glycerol-3-phosphate 3-phosphatidyltransferase (Fig. 9b and 10b). The key factor for PS biosynthesis promotion was not only the increased expression of enzymes related to the PS biosynthesis pathway but also enough substrates of CDP-DG and serine because of the upregulated expression of genes related to glyoxylate reductase (catalyzes glucose into serine) (43) and serine hydroxymethyltransferase (catalyzes glycine into serine) (Fig. 9b and 10b). Third, conversion of PE from PS has been promoted (Fig. 6a and c) due to the upregulated expression of the genes (*PSD1* and *PSD2*) and proteins [encoding phosphatidylserine decarboxylase (converts PS into PE) in *Monascus* (Fig. 9b and 10b] (44). Finally, insignificant difference in PC contents was a result of the balance between PC biosynthesis, which may be due to co-regulation of the CDP-DG pathway [upregulated expression of gene and protein related to phosphatidylethanolamine N-methyltransferase (catalyzes PE into PC) (45)] and the Kennedy pathway [upregulated expression of gene and protein related to choline phosphate cytidyl transferase (catalyze choline into PC)] (Fig. 9b and 10b), and hydrolysis (46), which may be due to the upregulated expression of genes and proteins related to lysophospholipase (conversion of PC to choline glycerophosphates) and lysophospholipase NTE1 [complete degradation of lysophospholipids to fatty acids, glycerides, and phospholipid acyl groups (14)] (Fig. 9b and 10b).

## MATERIALS AND METHODS

### *Monascus* culture

Stock cultures of the *Monascus purpureus* (CICC 5013), which was bought from the China Center of Industrial Culture Collection, were cultured on wort agar slants (containing 5° Bé wort and 15 g/L agar). Spore suspension (0.3 mL) was inoculated in 250 mL at 31℃ with continuous shaking at 200 rpm. Erlenmeyer flasks containing 30 mL of seed culture medium composed of 70 g/L starch (0, 5, 10, 15, and 20 g/L) NH_4_Cl, 5 g/L KH_2_PO_4_, 20 g/L glucose, 0.1 g/L CaCl_2_, and 0.7 g/L MnSO_4_. The sixth day of the control sample (0 g/L NH_4_Cl) and treated sample (15 g/L NH_4_Cl), each containing three biological replicates, were analyzed.

### Analysis of membrane integrity and permeability in *Monascus*

The integrity/permeability of *Monascus* cell membrane was analyzed with propidium iodide probe (47)/O-nitrobenzene-β-D-galactoside (ONPG) (48). The cell integrity was determined by the fluorescence intensity of the supernatant using a G-98000A fluorospectrophotometer (Agilent, USA) with 535 nm of excitation and 617 nm of emission. Membrane permeability of *Monascus* was analyzed with a ONPG probe as follows: logarithmic stage *Monascus* cells were collected, resuspended in M9 lactose induction medium for 24 h at 30℃, and 10 µL of ONPG (30 mmol/L) was added to 10 mL of *Monascus* suspension. Membrane permeability was determined by measuring the absorbance of the supernatant using a UV-1900PC spectrophotometer (Ao Yi Instrument Co., Ltd, Shanghai, China) at 420 nm.

### Analysis of membrane pH gradient (△pH) and transmembrane potential (△Ψ) in *Monascus*

An assay of △pH/△Ψ was carried out using a fluorescent probe of BCECF-AM/DiSC_3_(5) according to our previous report (48) as follows: Solution (5 mmol/L) of BCECF-AM/DiSC_3_(5) was prepared with DMSO. The *Monascus* precipitate collected by centrifugation at 8,000 *g* for 5 min was rinsed twice with 50 mM HEPES buffer (including 0.6 mmol/L KCl, 0.2% glucose and adjusted with 40% KOH to pH 7.0). HEPES buffer was added to 50 mL, and the fluorescent probe solution of BCECF-AM/DiSC_3_(5) was added to a final concentration of 5 mol/L and then incubated for 5/3 min at 30℃. The supernatant was centrifuged at 8,000 *g* for 5 min and filled to 3 mL with 50 mmol/L phosphate buffer (pH 6.0). G-98000A fluorospectrophotometer was used for analysis with 430/622 nm of excitation and 515/670 nm of emission.

### Determination of pigments, citrinin, ergosterol, Ca^2+^, and MDA in *Monascus*

Extraction of intracellular and extracellular pigments from *Monascus* was carried out according to previous reports (48). The pigment concentration was determined by the corresponding absorbance (410, 470, and 510 nm for yellow, orange, and red pigments, respectively) using a spectrophotometer (Unicosh Scientific Instrument Co., Ltd, Shanghai, China) and calculated by multiplying the absorbance values by the dilution factor according to the previous report (10). The linear equation between absorbance (*y*) and diluting proportions (*x*) is *y* = −0.0054*x* + 1.5462 (*R*^2^ = 0.9907, in the range from 100 to 300).

The determination of citrinin has been carried out using high-performance liquid chromatography (HPLC) method according to the previous report (11) as follows: an Agilent 1200 system (Santa Clara, CA, USA) equipped with a reverse-phase C18 column (ZORBAX SB-C18, 4.6 mm × 250 mm, 5 µm) was used. The mobile phase was composed of 75% solvent A (acetonitrile) and 25% solvent B (water with pH 2.5 adjusted by orthophosphoric acid) with a column temperature of 30°C at the flow rate of 0.8 mL/min. Fluorescence detection at 331 nm excitation wavelength and 500 nm emission wavelength was employed (Agilent 1260 Infinity II Multi-Wavelength Fluorescence Detector). A citrinin standard compound (Sigma, USA) was used to confirm the HPLC analysis.

The content of ergosterol was determined by the HPLC method according to the previous report (48) as follows: C18 column (4.6 ID × 250 mm, 5 µm), sample of 10 µL, mobile phase: 100% methanol; flow rate: 1.0 mL/min; column temperature: 30℃; and detection wavelength: 270 nm. Standard ergosterol was purchased from Beijing Solarbio Science & Technology Co., Ltd, China. The linearity equation *y* = 13,161*x* − 2,872.5 (*R*^2^ = 0.9998), where *y* is absorbance and *x* is dilution proportions (in the range from 100 to 300).

The Ca^2+^ concentration was determined by measuring the absorbance of the supernatant using a G-98000A fluorospectrophotometer at excitation 494 nm and emission 516 nm according to the previous report (48) as follows: the collected *Monascus* precipitate was supplemented once with Fluo-4 AM (0.5–5 μM) and probenecid (0.1–0.25 mM) to improve the staining efficiency and reduce Fluo-4 AM leakage and then incubated at 30℃ for 30 min; the solution was washed three times with HEPES buffer and incubated with 500 µL of HEPES buffer at 37℃ for 20 min.

The content of malonaldehyde in *Monascus* was assayed using the assay kit (manufactured by Nanjing Jiancheng Bioengineering Institute, China and Beijing Solarbio Science & Technology Co., Ltd, China) according to the manufacturer’s instructions.

### Absolute quantitative lipidomics analysis

*Monascus* mycelia of *Monascus* cultured in 0 and 15 g/L ammonium chloride collected by centrifugation at 8,000 *g* for 5 min were weighed for lipidomics analysis with UHPLC Nexera LC-30A system (Shimadzu) coupled to Q-Exactive Plus mass spectrometer (Thermo Scientific). Lipids were extracted according to the methyl tert-butyl ether method according to the previous report (48) as follows: 20 mg of collected *Monascus* mycelia was homogenized in 1.5 mL of chloroform/methanol (2:1, vol/vol), vortexed for 1 min, centrifuged at 3,000 rpm for 10 min, added to 800 µL organic phase in a clean tube, and dried with nitrogen. Sample preparation processes were performed in accordance with the above method of parallel preparation of quality control samples. Mass spectrometric analysis was conducted by adding 200 µL isopropanol/methanol solution (1:1, vol:vol), and the supernatant was used for analysis. Data analyses were performed according to the instructions of Shanghai Applied Protein Technology.

### TMT quantitative proteomics analysis

TMT quantitative proteomics analysis was performed by Shanghai Applied Protein Technology Co., Ltd. (China) according to the previous report (48) as follows: first, the collected *Monascus* mycelia were put into SDT lysis buffer (4% SDS, 100 mM Tris-HCl, and 1 mM DTT, pH 7.6), and the protein fraction was extracted. The protein levels in the supernatant were quantiﬁed with a BCA protein assay kit. An appropriate amount of protein was taken from each sample and trypsin digestion was performed by filter-aided proteome preparation, and then the peptide levels were quantiﬁed (OD_280_). Next, 100 µg peptide from each sample was labeled using TMT Isobaric Mass Tagging Kits (Thermo Scientific). The labeled peptides in each sample were mixed in equal amounts and graded using a High pH Reversed-Phase Peptide Fractionation Kit according to the manufacturer’s protocol. LC-MS/MS was performed on a Q-Exactive mass spectrometer (Thermo Scientific, USA) that was coupled to an Easy-nLC 1000 instrument (Thermo Scientific, USA). The TMT quantitative proteomics data have been deposited to the ProteomeX change Consortium via the PRIDE (49) partner repository with the data set identifier PXD042117.

### Validation of gene expression level by RT-qPCR

In order to analyze the genomic variation in *M. purpureus* related to the biosynthesis of lipids, ergosterol, and MPs, RT-qPCR was performed for 14 annotated genes involved in MP biosynthesis, 7 annotated genes involved in TG biosynthesis, 16 annotated genes involved in glycerophospholipids biosynthesis, and 8 annotated genes involved in ergosterol biosynthesis. For the removal of residual genomic DNA, RNA samples were treated with RNase-free DNaseI (Thermo Fisher Scientific, MA, USA) following the manufacturer’s protocol. The first-strand cDNA was synthesized using oligo-dT primers and *EasyScript* Reverse Transcriptase (TransGen Biotech, Beijing, China) according to the manufacturer’s protocol. RT-qPCR was performed using the *TransStart* Green qPCR SuperMix UDG (TransGen Biotech, Beijing, China) according to the manufacturer’s instructions. 2^-△△CT^ was used to determine the expression levels of the tested genes. The analysis used β-actin as the reference gene and PrimerQuest Tool was used for primer design, and the relevant primer sequences are shown in Tables 1 and 2.

**TABLE 1:** Primers used for RT-qPCR of *Monascus* pigment biosynthesis

Primer	Sequence	Primer	Sequence
actin-F	TTCGAGACCTTCAACGCCC	MpigG-R	CCACAACATCCTTCGTCTTGA
actin-R	ACCCTCGTAGATGGGAACGA	MpigH-F	CCAGGGCCGCAAGTTTAT
MpigA-F	AAGGGCCTCTTGACCTTTG	MpigH-R	GATCCACGAGACGAAGAAGATG
MpigA-R	CCTGAGACGAGTTCTTCGTATTT	MpigI-F	CATCAGTGGACGCTGCATAA
MpigB-F	GACCACGGTGCTGTATCTATG	MpigI-R	TCGTAGTGCCCAGGAAGAA
MpigB-R	CTAAAGACCGCTGCTCATACTC	MpigJ-F	GAGAAGGAACAGCGTGGATTAC
MpigC-F	GAGGCCCTGATGGTTTAAGT	MpigJ-R	CCGATCTTGGACTCAGGATAGA
MpigC-R	CGTTTCTCGGAGGTGAGATAG	MpigK-F	AGGCTGATCTCTTCTCTCCTAC
MpigD-F	CGACCCGTCTGTCAAGTTTAT	MpigK-R	CCTGCTCAACTTGGTCTCTATC
MpigD-R	TCATCTTGCACAGGTCATCC	MpigM-F	GAGAAGACACCAGAGGAAGTTATT
MpigE-F	CAGACTTTCCATGGGAGACATC	MpigM-R	CTCAGAAAGACGACGGAAGAC
MpigE-R	CTGCACTTCGGTCAGGTTATAC	MpigP-F	CGCAGAAACGCTCGACTATTA
MpigF-F	TGGACCAGGTCGAGAAGTAT	MpigP-R	GTAGGGAGATGGCTGTAAACTG
MpigF-R	CGATCTTCTGGAGGCTGTATTT	MpigQ-F	CGCAGCTAGGCATTGTCATA
MpigG-F	CTGGAGGAGCATCGGAAAC	MpigQ-R	GAAGAACAAGAGCAGACCTACC

**TABLE 2:** Primers used for RT-qPCR of lipid metabolic pathway

Primer	Sequence	Primer	Sequence
MPDQ_004118-F	TCCACGATTTGTACCACTGTC	MPDQ_005851-F	CAAGGAGCGATGGGAAGATAAA
MPDQ_004118-R	ACGACAACATCGGCTATTCC	MPDQ_005851-R	ACTGTCATCGGCATCGTATTG
MPDQ_002537-F	AGAACATGCTGTGGTGTATGG	MPDQ_007687-F	GGCGACTTCTTCTTCCTCATT
MPDQ_002537-R	GTGATGGTGCGTTGTGATTTG	MPDQ_007687-R	CAGCATAGCCGACAGAATACA
MPDQ_001197-F	CGGGTACGCTGATACCTTTAC	MPDQ_006892-F	CTTTGGAAGACCAGCCATACT
MPDQ_001197-R	GCTTCAATCGGAACAGGTTTG	MPDQ_006892-R	GTCACACCGAGAGCATACATAC
MPDQ_007484-F	CGTCAGGCTCATGACATCTT	MPDQ_006516-F	CTACGAGTGATGTGCTCAGTTT
MPDQ_007484-R	CGGTGCCAATCTTTCCAATAAA	MPDQ_006516-R	GACAACGCCATCTTTCATCTTG
MPDQ_003956-F	AGCCAGAAGAGCGGAATATG	MPDQ_002295-F	CCACCACTCACAAGTCTCTTC
MPDQ_003956-R	GAAGCGGCAGAAATGGAAAG	MPDQ_002295-R	CATGATCTCCTTACCGGTCTTG
MPDQ_007056-F	CTCGTTCTGGACCGCAATTA	MPDQ_005352-F	GTGGTAGAAGAAGCAGGAACA
MPDQ_007056-R	TCTGGTTTGTGGGACTTTCTC	MPDQ_005352-R	CCGGATGAGAAGAGGTCAAAG
MPDQ_000757-F	CCATGGCAGGAACTCAAGAA	MPDQ_006876-F	CACTAGAATCGTGCGTGACTAC
MPDQ_000757-R	AGAACACATCGCAGGCATAG	MPDQ_006876-R	CACTTCCAGCTCGTTCTTCTT
MPDQ_005305-F	GCCATTGCACCCTCTATCTT	MPDQ_001434-F	ACTCTCATCGGCAGCAATAC
MPDQ_005305-R	GTCGTATCCCAGACCCAATAAA	MPDQ_001434-R	GAGGTATTCGAGAGGAGCAAAG
MPDQ_007694-F	GAGTGATTGCGTCGGTAGTT	MPDQ_006976-F	TTGACGTATCCCACGGTTTC
MPDQ_007694-R	TGGTATATCGCATGACCCATTT	MPDQ_006976-R	CCAGCCAGAAATCCTCAATCT
MPDQ_001115-F	CAGGGAAGGAGAACCCATTT	MPDQ_006926-F	TTGTCAGCCATCAGCGTATC
MPDQ_001115-R	TCCAAAGATACCTCCGCATTAG	MPDQ_006926-R	GCAACGGTCTCCGGATTATT
MPDQ_002346-F	GCGGGTATGATGTTCCAGATA	MPDQ_001761-F	TACTCCGGAGAATGAGAAGGA
MPDQ_002346-R	CTTGCTGACACCAGAAGATAGA	MPDQ_001761-R	CCTGGACACCTCTCTTGATAAC
MPDQ_003109-F	GTGCTTGGCTTCCTCTATGT	MPDQ_004727-F	TGCGTGGCTTGTCAGATATT
MPDQ_003109-R	AGCTGCCTTCTCAGTTTCTC	MPDQ_004727-R	CCCATTGGGAAGACTGTATGAA
MPDQ_002604-F	TCGCATCAAGTCCCGATTAC	MPDQ_003223-F	GACATCGGAAGAGGAGATGAAG
MPDQ_002604-R	GAGGCCCTTGTCTTCGTATTT	MPDQ_003223-R	AAAGAGCCCATGGTCAGTATC
MPDQ_005295-F	AGCCCATGCAGTTCCATATC	MPDQ_007823-F	CCTTCCAGGAATGGATGGTTAG
MPDQ_005295-R	AGACTCGCCGAAATCCTTTATT	MPDQ_007823-R	ACGTCGAGAGAGAAGGATAGAG
MPDQ_003516-F	GTCGGGCAAGATGATTGATTTC	MPDQ_005541-F	CCTGGTGGAAGCGAATTGA
MPDQ_003516-R	ACGACATAGGACCCAAGAGA	MPDQ_005541-R	CTCGTAAGCAGAGTCGGAATG
MPDQ_005533-F	CCCAGTTGCTCAGGATGATATG	MPDQ_006185-F	CACTTGCATTGGCGTGTATATG
MPDQ_005533-R	CGGGTGAAGTGGCATGTAATA	MPDQ_006185-R	GTCCCTTCATAAGGGCGATAAA
MPDQ_001807-F	GGCTCTTCACAGCAGGATATT	MPDQ_002328-F	AACTGGTGCGAAGAGGATTAC
MPDQ_001807-R	CTCGGTTGTGGATGTGGATT	MPDQ_002328-R	CCCTTGACTCCAAGCCATAC
MPDQ_002587-F	GGCCCTCATCTCCAACAAA	MPDQ_002948-F	CGAACGGTGGTCCAGTTATT
MPDQ_002587-R	AGGACCAAGGCGGTTATTG	MPDQ_002948-R	GGCTTTCCTAGCTTGGTCTATC
MPDQ_002113-F	GTTTCCCAAGGCTGGATTCT	MPDQ_004037-F	GTCCATCATCGTCGGTTCTATC
MPDQ_002113-R	CCATCAGGCAACCGTGTATAA	MPDQ_004037-R	CAAATGCCCACACTGAAACC
MPDQ_005530-F	CACACTTCCCACCCTTCTTT	MPDQ_003224-F	GTGAGCTGTTCCCATCCAAATA
MPDQ_005530-R	CCTCAGACTCAGAACTCCATTG	MPDQ_003224-R	GGAGATAAAGGGCGTGAAGAAG

### Statistical analysis

All data were evaluated using analysis of variance, and significant differences among the means of three replicates (*P* < 0.05) were determined by Turkey’s test using SPSS version 17.0 software (SPSS Inc., Chicago, IL, USA). All figures were drawn with OriginPro 8.0 (OriginLab Corporation, Northampton, MA, USA).

## Data Availability

All data generated or analyzed during this study are included in this published article and its supplemental files.

## SUPPLEMENTAL MATERIAL

Table S1 aem.01146-24-s0001.xls Difference in lipidIon in *Monascus* cultured with ammonium chloride between 0 g/L and 15 g/L.

Table S2 aem.01146-24-s0002.xls Difference in lipidclass in *Monascus* cultured with ammonium chloride between 0 g/L and 15 g/L.

Table S3 aem.01146-24-s0003.xlsx Significant difference analysis of protein expression in *Monascus* cultured with ammonium chloride between 0 g/L and 15 g/L.

ASM does not own the copyrights to Supplemental Material that may be linked to, or accessed through, an article. The authors have granted ASM a non-exclusive, world-wide license to publish the Supplemental Material files. Please contact the corresponding author directly for reuse.
